# Long-Term Stable Mixed Chimerism after Hematopoietic Stem Cell Transplantation in Patients with Non-Malignant Disease, Shall We Be Tolerant?

**DOI:** 10.1371/journal.pone.0154737

**Published:** 2016-05-06

**Authors:** Arwen Stikvoort, Mikael Sundin, Mehmet Uzunel, Jens Gertow, Berit Sundberg, Marie Schaffer, Jonas Mattsson, Michael Uhlin

**Affiliations:** 1 Department of Oncology-Pathology, Karolinska Institute, Stockholm, Sweden; 2 Centre for Allogeneic Stem Cell Transplantation (CAST), Karolinska University Hospital, Stockholm, Sweden; 3 Division of Paediatrics, Department of Clinical Sciences, Intervention and Technology (CLINTEC), Karolinska Institute, Stockholm, Sweden; 4 Hematology/Immunology/HSCT Section, Astrid Lindgren Children’s Hospital, Karolinska University Hospital, Stockholm, Sweden; 5 Department of clinical immunology and transfusion medicine, Karolinska University Hospital, Stockholm, Sweden; University of Lisbon, PORTUGAL

## Abstract

Long-term stable mixed chimerism is a rare and poorly understood phenomenon post hematopoietic stem cell transplantation. This study aims to shed light on whether the two hematopoietic systems in patients with mixed chimerism remain functional. Additionally, we investigate possible immunologic differences in these individuals compared to patients with only donor derived immune cells. Patients with donor and mixed chimerism, at median 10 (5–16) years post-HSCT for non-malignant diseases, were assessed regarding clinical situation and immune system (phenotypical and functional). No difference in long-term outcome was seen in terms of general wellbeing, central phenotypic immune system features (*e*.*g*., differentiation status, CD4/CD8 ratio, B and NK-cell frequency) and antibody responses to immunizations. At a median of 10 years post transplantation, patients with mixed chimerism had significantly higher IgG3 and platelet levels. Additionally, these patients had higher NKT-cell levels (CD94+CD8+ and CD56+CD8+) than patients with donor chimerism. In depth phenotypic analysis of patients with mixed chimerism demonstrated recipient-derived fractions in most immune cell lineages (*e*.*g*., T-cell, B-cell and NK-cell subsets). Recipient cells were also capable of responding to mitogenic stimulation with production of several cytokines. In conclusion, long-term mixed chimerism did not negatively affect patient wellbeing and long-term outcome. Moreover, recipient-derived immunity may still be functional in these patients, suggesting an active state of tolerance and immunologic dependence on both hematopoietic systems.

## Introduction

Hematopoietic stem cell transplantation (HSCT) is an established curative treatment for several genetic, metabolic and hematologic disorders.[1–3] Patients receive and adopt a donor hematopoietic system after their own system has been compromised by a conditioning regimen including chemotherapeutic agents and/or irradiation.[4] Eventually, full donor chimerism (DC) is achieved when the new donor hematopoietic system completely replaces the recipient system.

As it is not necessary to achieve a graft-versus-leukaemia (GvL) effect in patients with non-malignant disorders, reducing the chance of graft-versus-host disease (GvHD) development in these patients is important.[2, 5–8] Therefore, patients with non-malignant diseases tend to be given less intensive conditioning regimens than patients with malignancies.[9] Due to the intensity-reduction, patients are consequently at a higher risk for graft rejection, often demonstrated by an increase of detectable recipient derived cells.[10] DC is achieved when more than 95% of the hematopoietic system consists of donor-derived cells. However, a small subset of patients never reaches full DC, which is referred to as mixed chimerism (MC). MC is defined as having 5–95% recipient-derived hematopoietic cells remaining.[11]

An increase of recipient derived hematopoietic cells can be interpreted as threatening rejection (or relapse in patients with malignancies), but not necessarily so in all patients.[10, 12] Development of high-grade, progressive MC can be treated with donor lymphocyte infusions (DLIs) in an effort to boost the donor-derived immunity. Unfortunately, DLIs have been associated with the occurrence of GvHD, occasionally deleterious.[13, 14]

This raises the question whether it is necessary to treat MC development in patients with non-malignant diseases. To answer this, MC development needs to be studied further, especially in the rare instances where it has developed into long-term stable MC without complications for several years post-HSCT.[15, 16] Most patients with long-term stable MC have a recipient-donor chimerism, though donor-donor chimerism after double cord blood transplantation (DCBT) has been observed.[17, 18]

The mechanisms driving MC development are still largely unknown; however, a previous publication from our group suggested a role for donor type.[19] Human Leukocyte Antigen (HLA) matched sibling donors appeared to have a significant positive impact on MC development. Additionally, lower incidence of both acute GvHD and blood stream infections occurred in these patients. Not much is known whether the remaining immune cells from the recipient are functional and if there are differences between individuals with DC or MC. The present study provides a more in-depth analysis and comparison of the phenotypic and functional features of the hematopoietic systems in patients with DC and MC.

## Material and Methods

### Patients

Twelve patients with long-term stable MC were matched with thirteen DC patients, all with non-malignant disorders, at median 10 years post-HSCT. The HSCTs were performed between 1996 and 2007 at the Centre for Allogeneic Stem cell Transplantation, Karolinska University Hospital, Huddinge, Sweden. Every consecutive MC patient transplanted between 1996 and 2007 with non-malignant disease and still alive in 2012 was selected for the study. DC patients were selected for the same criteria and matched for age and gender. Patient characteristics (Table 1) are found in the results section, additional patient details were published previously.[19] Patients are referred to by their Unique Patient Number (UPN) or unique symbols (Table 1) throughout the article. S1 Table depicts an overview of patients for whom samples were available for each method. Written informed consent was obtained of all patients, or their parents in the case of minors, before enrolment in the study. The study was approved by the Regional Ethical Review Board in Stockholm, Sweden and performed according to the amended Declaration of Helsinki.

**Table 1:** Patient Characteristics.

Chimerism status	UPN	Symbol in graphs	HSCT year	Age at HSCT (rec/don)	Diagnosis	Conditioning regimen	Drugs used for conditioning	Donor type	Stem cell source	Anti T-cell antibody treatment	Lansky/ Karnofsky
**MC**	527	✳	1996	21/18	SAA	RIC	Cy	Sibling	BM	Thymoglobulin	90
	539	NA	1996	3/5	SAA	RIC	Cy	Sibling	BM	ATG-Fresenius S	100
	603	✶	1997	5/28	AGU	MAC	fTBI+Cy	MUD	PB	Orthoclone OKT-3	100
	615	+	1997	14/6	SAA	RIC	Cy	Sibling	BM	Orthoclone OKT-3	100
	652	✕	1998	7/1	β-thalassemia major	MAC	Bu+Cy	Sibling	CB+BM	Thymoglobulin	90
	906	⨀	2000	22/25	SAA	RIC	Cy	Sibling	BM	Thymoglobulin	100
	921	⚀	2002	9/12	SAA	RIC	Cy	Sibling	BM	Thymoglobulin	100
	1012	NA	2004	11/50	Fanconi anaemia	RIC	Flu+Cy	MUD	BM	Thymoglobulin	100
	1098	⨂	2005	13/2	β-thalassemia major	MAC	Bu+Cy	Sibling	BM	Thymoglobulin	100
	1112	☒	2005	8/0	Fanconi anaemia	RIC	Flu+Cy	MUD	CB	Thymoglobulin	90
	1208	⟐	2007	16/19	CGD	RIC	Flu+Treo	Sibling	BM	Thymoglobulin	90
	1240	#	2007	6/6	β-thalassemia major	MAC	Bu+Cy	Sibling	BM	-	90
**DC**	628	⚫	1998	1/30	WAS	MAC	Bu+Cy	MUD	BM	ATG-Fresenius S	90
	707b	⬛	2000	40/29	ALD	RIC	Flu	MUD	BM	Thymoglobulin	80
	731	▲	1999	9/42	SAA	RIC	fTBI+Cy	MUD	BM	Thymoglobulin	100
	822	NA	2001	2/17	Sickle cell anaemia	MAC	Bu+Cy	Sibling	BM	-	100
	887	▼	2002	37/39	SAA	RIC	Flu+fTBI+Cy	MUD	BM	Thymoglobulin	100
	909	◆	2002	6/21	SAA	RIC	Flu+fTBI+Cy	MUD	BM	Thymoglobulin	100
	954	NA	2003	8/41	Fanconi anaemia	RIC	Flu+Cy	MUD	BM	Thymoglobulin	100
	955	○	2003	38/43	SAA	RIC	Flu+fTBI+Cy	MUD	BM	Thymoglobulin	100
	1065	⬜	2004	11/31	SAA	RIC	Flu+fTBI+Cy	MUD	BM	Thymoglobulin	100
	1111	NA	2005	13/17	Sickle cell anaemia	MAC	Bu+Cy	Sibling	BM	-	100
	1166	△	2006	9/40	SAA	RIC	Flu+fTBI+Cy	MUD	PB	Thymoglobulin	90
	1167	▽	2006	9/41	SAA	RIC	Flu+fTBI+Cy	MUD	BM	Thymoglobulin	100
	1229	◇	2007	11/0	CGD	RIC	Flu+Treo	MMUD	CB	Thymoglobulin	100

MC = Mixed Chimerism; DC = Donor Chimerism; UPN = Unique Patient Number; NA = Not Applicable; HSCT = Hematopoietic Stem Cell Transplantation; rec = Recipient; don = Donor; WAS = Wiskott-Aldrich Syndrome; ALD = Adrenoleukodystrophy; SAA = Severe Aplastic Anaemia; CGD = Chronic Granulomatous Disease; AGU = Aspartylglucosaminuria; MAC = Myeloablative Conditioning; RIC = Reduced Intensity Conditioning; MUD = Matched Unrelated Donor; MMUD = Mismatched Unrelated Donor; BM = Bone Marrow; PB = Peripheral Blood; CB = Cord Blood; Cy = Cyclophosphamide; fTBI = fractionated Total Body Irradiation; Bu = Busulfan; Flu = Fludarabine; Treo = Treosulphan

### Questionnaires

Patients completed an in-house questionnaire, based on a study by Winterling *et al* (2014), regarding their general and medical wellbeing over the past 5 years.[20] Questions varied from occurrence of diarrhoea, fever, sinopulmonary infections, skin problems, use of antibiotics, use of other medical drugs, sick leave and ability to work/study fulltime (S2 Table).

### Sample preparation

Blood samples were drawn at median 10 (5–16) years post-HSCT. In addition, plasma samples were selected for the patients at day 14 post-HSCT for a better indication of immune-phenotype close to HSCT. Plasma was separated from blood samples (500g, 10 min; Rotina 420 [Hettich, Beverly, MA, USA]) and stored at -80°C. Peripheral blood mononuclear blood cells (PBMCs) were separated by density gradient centrifugation (800g, 20 min; Lymphoprep [Fresenius Kabi, Oslo, Norway]) and frozen at -196°C in 10% DMSO in complete RPMI-1640 medium (HyClone® [Thermo Fisher Scientific Inc., Waltham, MA, USA]), enriched with 10% fetal calf serum (FCS [Gibco, Life Technologies, Paisley, UK]) or 10% human AB-serum [Karolinska University Hospital], 2 mM L-Glutamine [Gibco], 100 IU/ml penicillin G [Gibco], 100 mg/ml streptomycin [Gibco], 1% HEPES [Sigma-Aldrich, St. Louis, MO, USA], 1% non-essential amino acids (MEM [Sigma-Aldrich]) and 1% Sodium Pyruvate [Sigma-Aldrich].

### DNA purification

DNA was purified according to manufacturer’s protocol with a QIAamp DNA mini kit [Qiagen, Hilden, Germany], with two additional steps. To improve DNA yield, 1μl carrier RNA [Qiagen] was added at the same step as Buffer AL. Additionally, preheated (56°C) distilled H_2_O was used to elute the DNA. DNA concentration was assessed using a NanoDrop 2000 spectrophotometer [Thermo Fisher Scientific Inc.]. DNA was stored at -20°C.

### Human Leukocyte Antigen typing

HLA-typing was performed using either PCR-SSO on a Luminex platform (One Lambda, Ca, USA) for low resolution, or low and high-resolution using PCR-SSP (Olerup SSP, Stockholm, Sweden).[21]

### Immunonephelometric and ELISA assay

Plasma IgG and IgG subclasses were assessed by nephelometric assays as described previously.[22, 23] Antibody concentrations against immunization antigens (i.e., *C*. *tetani*, *H*. *influenzae*, *S*. *pneumoniae* and *C*. *diphtheriae*) were determined using an Enzyme Linked Immuno Sorbent Assay (ELISA) assay as described previously.[24] Both assays were analysed on a Dade Behring BN™II Nephelometer [Dade Behring Canada Inc., Mississauga, ON, USA].

### Multiplex assay

Plasma levels of 26 different cytokines (Eotaxin, G-CSF, GM-CSF, IFN-α2, IFN-γ, IL-10, IL-12 (p40), IL-12 (p70), IL-13, IL-15, IL-17, IL-1α, IL-1β, IL-2, IL-3, IL-4, IL-5, IL-6, IL-7, IL-8, IP-10, MCP-1, MIP-1α, MIP-1β, TNF-α and TNF-β) were determined using the MILLIPLEX MAP Human Cytokine/Chemokine—Premixed 26 Plex from Millipore [Millipore Corporation, Temecula, CA, USA] according to manufacturer’s protocol and as described before.[25–27] Analysis was done with the Luminex IS 2.3 software [Luminex Corp., Austin, TX, USA] on the LABScan100 (One Lambda Inc., Canoga Park, CA, USA).

### Chimerism analysis

All 25 patients were analysed for chimerism status, as previously described.[19] The first chimerism status was determined for T-cells (CD3+), B-cells (CD19+) and myeloid cells (CD33+). Nine out of twelve patients classified as MC were further analysed for chimerism in the CD4+ T-cell, CD8+ T-cell, NK-cell (CD56+CD3-), TCRγδ+ T-cell lineages and for cytokine-producing lymphocytes in response to mitogenic stimulation.

Chimerism analysis, based on variable number tandem repeats [28], on short tandem repeats [29] and on single nucleotide polymorphisms (SNPs) [30], was used to determine the percentage of recipient-derived cells. The ABI 700 Sequence Detection System [Applied Biosystems, Foster City, CA, USA] and the 3130xl genetic analyser for capillary electrophoresis [Applied Biosystems] were used for detection and quantification.[11]

### Mitogenic stimulation assay

PBMCs were incubated for 4 hours (5% CO_2_, 37°C) in complete RPMI-1640 medium [HyClone®, Thermo Fisher Scientific Inc.] enriched with 10% human AB serum, 1% L-glutamine and 1% penicillin-streptomycin (PEST). Samples were incubated in 2 conditions; a non-stimulation control condition (enriched RPMI-1640 medium with 10μg/ml Brefeldin A (BFA; [Sigma-Aldrich])) and a stimulation condition (enriched RPMI-1640 medium with 10μg/ml BFA, 25ng/ml phorbol 12-myristate 13-acetate (PMA; [Sigma-Aldrich]) and 1μg/ml Ionomycin [Sigma-Aldrich]).[31] PBMCs were incubated at a concentration of 4×10^6^ cells/ml medium in flat-bottomed well plates. After incubation, PBMCs were used for Fluorescence-Associated Cell Sorting (FACS), described below.

### Western blot

Western blot was performed as previously described.[32] The antibodies and reagents used are described in the next section. Imaging was done on a Fuji Intelligent Dark Box II with LAS-1000 software [Fuji, Tokyo, Japan] and Bio-rad FluorS MAX MultiImager with Bio-Rad Quantity One software [Bio-Rad Laboratories Inc., Hercules, CA, USA]. Images were analysed with ImageJ software [National Institutes of Health, Bethesda, MD, USA]. Intensities of protein-specific bands were calculated relative to respective Actin intensity to account for loading irregularities.[32]

### Western blot antibodies and reagents

The anti-phosphoserine (4A4) and anti-phosphotyrosine (4G10) mouse monoclonal antibodies (mAbs) [Millipore Corporation]; and CD3-ζ chain (6B10.2), LCK (3A5) and ZAP-70 (SB70) mouse mAbs [Santa Cruz Biotechnology, Inc, Santa Cruz, CA, USA], were used in a 1:1000 dilution. Anti-Actin mouse mAb (AC-40) [Sigma-Aldrich] and goat anti-Mouse-horseradish-peroxidase (HRP) conjugate (cat. # 170–5047) antibody [Bio-Rad], were used in a 1:2000 dilution.

### Flow cytometry

PBMC staining was performed as previously described.[18] The antibodies and reagents used are described in the next section. For intracellular staining the protocol of the Foxp3/Transcription Factor Staining Buffer [eBioscience Inc.] or the BD Cytofix/Cytoperm™ kit [BD Biosciences] was used. Acquisition was performed with the BD LSRII using BD FACS Diva software [BD Biosciences], the Beckman Coulter Gallios using Beckman Coulter Gallios software [Beckman Coulter] or the BD FACS Aria using BD FACS Diva software [BD Biosciences]. Sorting was done with the BD FACS Aria using BD FACS Diva software. Analysis was done with FlowJo software [Tree Star, Inc., Ashland, OR, USA]. Fluorescence-minus-one (FMO) samples were used to obtain proper gating strategies.[33]

### Flow cytometry antibodies and reagents

Fluorescein isothiocyanate (FITC) anti-CD3 (SK7); FITC anti-CD19 (HIB19); FITC anti-CD28 (CD28.2); FITC anti-CD45RO (UCHL1); FITC anti-CD56 (NCAM16.2); FITC anti-CD69 (FN50); FITC anti-CD94 (HP-3D9); FITC anti-CD95 (DX2); FITC anti-TCR αβ (WT31); phycoerythrin (PE) anti-CD3 (SK7); PE anti-CD25 (M-A251); PE anti-CD45RA (HI100); PE anti-CD56 (NCAM6,2), PE anti-IL-2 (MQ1-17H12); 7-Amino-Actinomycin D (7-AAD); PE-Cy5 anti-CD3 (UCHT1); PE-Cy7 anti-CD3 (SK7); PE-Cy7 anti-CCR7 (3D12); PE-Cy7 anti-IFNγ (B27); allophycocyanin (APC) anti-CD3 (SK7); APC anti-CD4 (RPA-T4); APC anti-CD8 (RPA-T8); APC anti-CD27 (L128); APC anti-CD45RO (UCHL1); APC-Cy7 anti-CD8 (SK1); Alexa Fluor 700 anti-CD4 (RPA-T4); V450 anti-CD3 (UCHT1) were purchased from BD Biosciences, San Jose, CA, USA. FITC anti-FOXP3 (236A/E7); APC anti-FOXP3 (236A/E7) and Alexa Fluor 700 anti-CD4 (OKT-4) came from eBioscience, Inc., San Diego, CA, USA. Qdot605 anti-CD3 (UCHT1) and Pacific Orange anti-CD8 (3B5) came from Invitrogen, Eugene, OR, USA. FITC anti-TCR PANγδ (IMMU510) and Krome Orange anti-CD4 (13B8.2) was purchased from Beckman Coulter, Immunotech, Marseille, France.

### Data and statistical analysis

Data was analysed and displayed with Excel 2011 [Microsoft Corp, Redmond, WA, USA] and Prism 5 [GraphPad, San Diego, CA, USA] software. Univariate statistical analysis was done with the Mann-Whitney-U test, Fisher’s exact test, Wilcoxon signed-rank test and Spearman’s rank correlation coefficient using Prism 5 software. Statistical significance was set at p<0.05, two-tailed. Data in tables are presented as median values and range (minimum-maximum) or as absolute numbers. In graphs, data are shown as concentrations, frequency of cells from parent cell subset, median values or as frequency of recipient cells.

## Results

### Patient characteristics

The study-specific questionnaires were completed by 23 of 25 patients (MC, n = 12 and DC, n = 11) and revealed no difference in life quality between patients with MC and DC at median 10 years post-HSCT (S2 Table). Both groups had a similar infection burden, medication usage and ability to work/study fulltime. Although no difference in infection incidence could be observed at median 10 years post-HSCT, shortly after transplantation an increased incidence of blood stream infections (BSI) was seen in the DC patient group (MC, n = 0/12 and DC, n = 5/13) [19].

Additionally, HLA typing demonstrated more HLA-C mismatches in the DC patient group (MC, n = 1/12 and DC, n = 3/12), though this difference was not significant. It is however consistent with our previous significant finding of more sibling donors in the MC patient group, where HLA-C mismatches are less common.[19] There were no differences in T-cell depletion strategies or in the use of RIC or MAC conditioning regimens. However, there were differences between the patient groups in terms of more specific conditioning components. In a previous paper we reported an association of cyclophosphamide alone with MC development and the use of fludarabine and fractionated total body irradiation with DC development.[19]

### Soluble characterization

A phenotypic characterization was performed on soluble factors in plasma. There was no significant difference found in cytokine concentrations 14 days post-HSCT between the two groups (MC, n = 4 and DC, n = 8; S3 Table).

**Fig 1:**
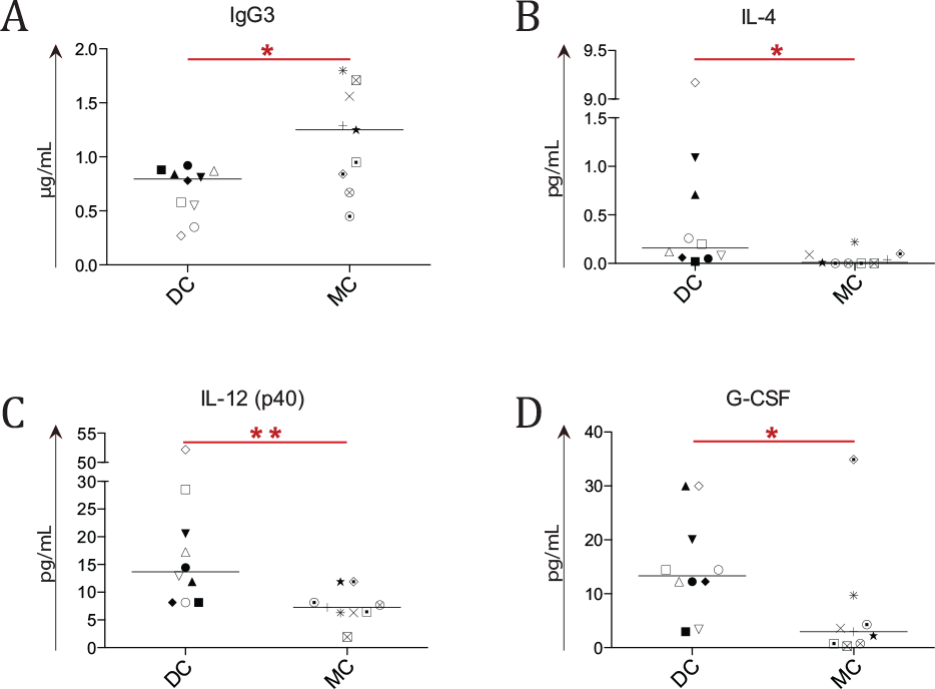
Comparison of soluble biomarkers between patients with mixed and donor chimerism. Concentrations of cytokines, IgG and IgG subclasses were determined in the plasma of 9 mixed chimerism (MC) and 10 donor chimerism (DC) patients at median 10 years post-HSCT. Asterisks indicate significant *P*-values (* = *P* < .05 and ** = *P* < .01), symbols indicate individual patient levels and horizontal bars in scatter graphs indicate median values of the patient group. (A) A higher IgG3 concentration was seen in MC patient plasma (*P =* .027). (C-D) A lower concentration of IL-4 (B), IL-12 (p40) (C) and G-CSF (D) was observed in MC patients (*P =* .016, *P =* .003 and *P =* .022 respectively).

After a median of 10 years post-HSCT (MC, n = 9 and DC, n = 10), patients with MC had higher IgG3 concentrations (*P =* .027, Fig 1A and S3 Table). No difference was observed for total IgG, IgG1, IgG2 and IgG4 levels (S1A–S1D Fig and S3 Table). Additionally, patients with MC were found to have lower IL-4, IL-12 (p40) and G-CSF concentrations (*P =* .016, *P =* .003 and *P =* .022, respectively; Fig 1B–1D and S3 Table). No difference was observed for immunization responses (i.e., specific IgG against *C*. *tetani*, *H*. *influenzae*, *S*. *pneumoniae* and *C*. *diphtheriae*; S1E–S1H Fig and S3 Table).

### Cellular characterization

**Fig 2:**
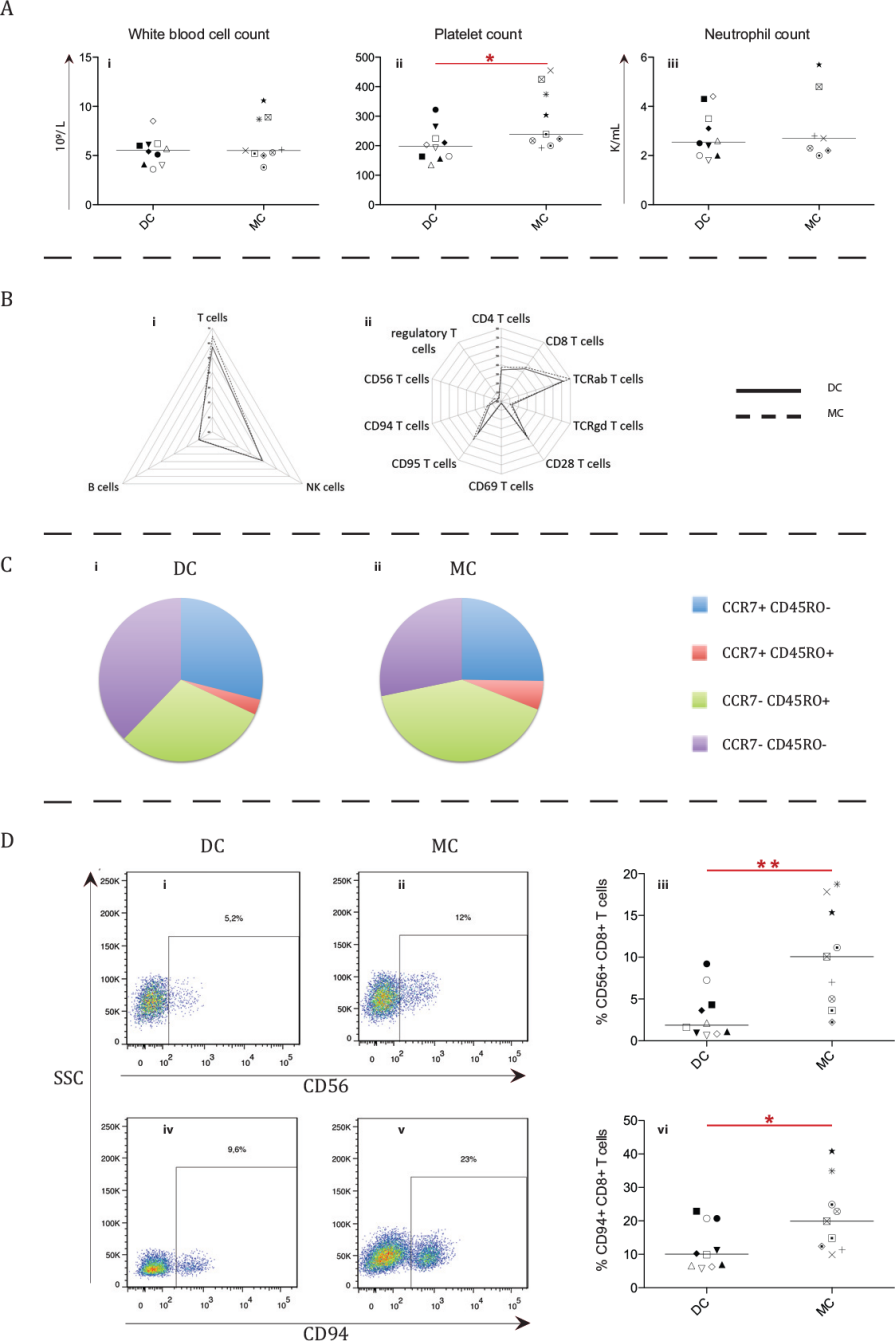
Phenotypic comparison of cellular subsets between patients with mixed and donor chimerism. For most cellular subsets no significant differences were observed between 9 mixed chimerism (MC) and 10 donor chimerism (DC) patients (A-C). Asterisks indicate significant *P*-values (* = *P* < .05 and ** = *P* < .01), symbols indicate individual patient levels and horizontal bars in scatter graphs indicate median values of the patient group. (A) The white blood cell (i), platelet (ii) and neutrophil (iii) count in the two patient groups. Platelet counts were higher in MC patients (*P =* .041). K/mL = 1 000 cells/mL. (B) Radar graphs depicting relative distribution of T, B and NK-cells (i) and T-cell subsets (ii) for DC and MC patient groups. (C) Differentiation status of total T-cells (CD3+), as defined by naïve memory (CCR7+ CD45RO-), central memory (CCR7+ CD45RO+), effector memory (CCR7- CD45RO+) and terminally differentiated memory (CCR7- CD45RO-), was found to be similar between the DC and MC patient groups. (D) Representative FACS plots of potential NKT-cells (CD56+ (i-ii) or CD94+ (iv-v)) gated on CD8+ T-cells. In the corresponding graphs (iii, vi)), individual ratios of the subsets for each group are shown (*P =* .004 and *P =* .035 respectively).

At median 10 years post-HSCT, few major cellular phenotypic differences were observed between patients with MC and DC (n = 9 and n = 10). White blood cell and neutrophil counts were similar, but patients with MC had higher platelet counts (*P =* .041; Fig 2Aii). Differentiation status of T-cells (CD3+), as defined by naïve memory (CCR7+CD45RO-), central memory (CCR7+CD45RO+), effector memory (CCR7-CD45RO+) and terminally differentiated memory (CCR7-CD45RO-), were similar in both patient groups (Fig 2C). No difference was observed for several T-cell subsets such as CD28+ and TCRγδ+ T-cells (Fig 2Bii). Additionally, frequencies of CD4+ T-cells, CD8+ T-cells, B-cells (CD19+CD3-) and NK-cells (CD56+CD3-) were similar in both groups (Fig 2B and S2 Fig).

However, patients with MC had an increased frequency of CD8+ T-cells expressing NK-cell associated markers CD56 (*P =* .004; MC = 10.1% versus DC = 1.9%) and CD94 on their surface (*P =* .035; MC = 19.9% vs. DC = 10.1%; Fig 2Diii and 2Dvi).

**Fig 3:**
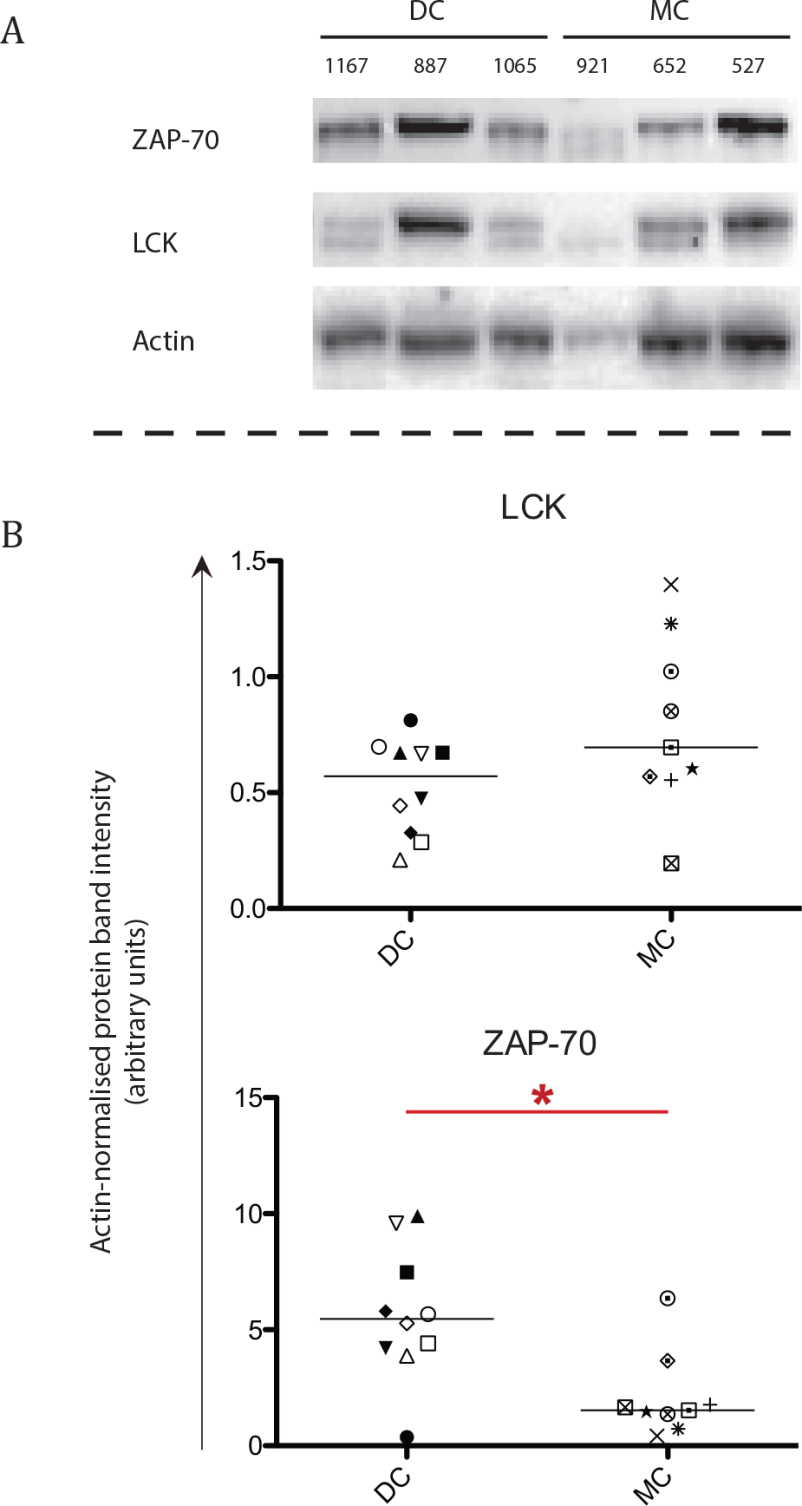
Protein expression of molecules involved in lymphocyte signalling between patients with mixed and donor chimerism. Protein expression of ZAP-70, LCK and actin was assessed in lymphocytes of 9 mixed chimerism (MC) and 10 donor chimerism (DC) patients. Asterisks indicate significant *P*-values (* = *P* < .05), symbols indicate individual patient levels and horizontal bars in scatter graphs indicate median values of the patient group. (A) Representative blots displaying 3 DC (UPN 1167, 887 and 1065) and 3 MC patients (UPN 921, 652 and 527). (B) The individual values for LCK and ZAP-70 of all patients, with regards to their respective actin intensity. A difference was observed for ZAP-70 expression (*P =* .013) between the DC and MC patient groups.

Western blotting (MC, n = 9 and DC, n = 10) was used to measure the relative protein expression of several signalling molecules in the total lymphocyte population (Fig 3A). Patients with MC had significantly lower expression of ZAP-70 (*P =* .013) in their lymphocytes than those with DC (Fig 3B). No differences were observed for LCK.

### Functional comparison

**Fig 4:**
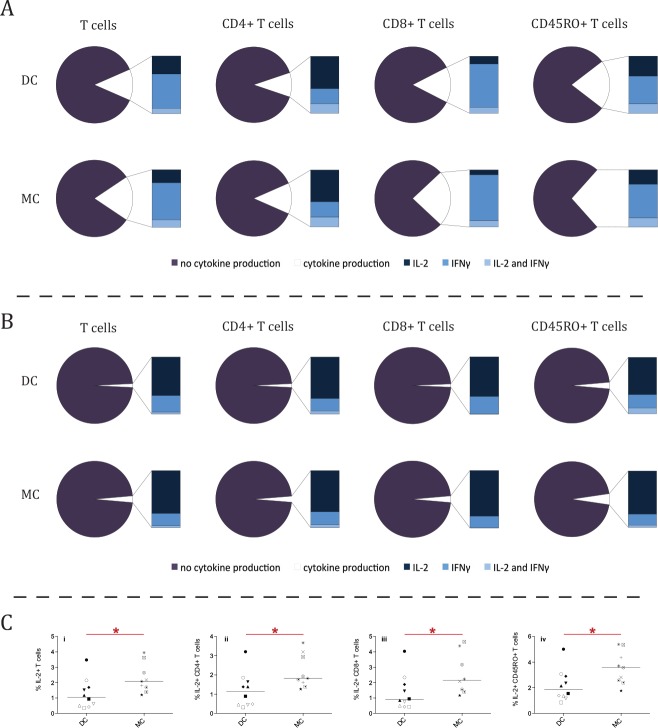
Higher frequency of IL-2-producing cells for steady state lymphocytes in mixed chimerism patients. The frequency of IL-2 producing cells after a 4 hour mitogenic stimulation of 9 mixed chimerism (MC) and 10 donor chimerism (DC) patients. (A) Median frequency of IL-2 and IFNγ producing lymphocytes of several T-cell (CD3+) subsets (total T-cells, CD4+ T-cells, CD8+ T-cells and CD45RO+ T-cells) in the mixed chimerism (MC, n = 9) and donor chimerism (DC, n = 10) groups after a 4-hour mitogenic stimulation with PMA and Ionomycin. No statistical difference was observed between the patient groups. (B) Pie charts displaying the median results for the same T-cell subsets after a 4-hour non-stimulation (simulating steady-state production of IL-2 and IFNγ). A higher frequency of IL-2 producing cells could be observed. (Ci-iv) The dot plots display a difference in the frequency of IL-2 producing cells between the DC and MC patient groups in the non-stimulation condition for the same T-cell subsets. The frequency was higher for total T cells (i), *P =* .017; CD4 T cells (ii), *P =* .034; CD8 T cells (iii), *P =* .034; and memory (CD45RO+) T cells (iv), *P =* .022. Asterisks indicate significant *P*-values (* = *P* < .05), symbols indicate individual patient levels and horizontal bars in scatter graphs indicate median values of the patient group.

A mitogenic stimulation assay was performed to investigate whether there were functional differences between patients with MC and DC (MC, n = 9 and DC, n = 10). While no differences in the frequency of IL-2 and IFNγ producing cells were observed after mitogenic stimulation (Fig 4A), there was a higher frequency of IL-2-producing cells in patients with MC in the non-stimulated condition (Fig 4B and 4C). Higher frequencies of IL-2-producing cells were detected in the total T-cell (*P =* .017), CD4+ T-cell (*P =* .034), CD8+ T-cell (*P =* .034) and CD45RO+ T-cell (*P =* .022) subsets (Fig 4C). No difference in Median Fluorescence Intensity (MFI) was seen for IL-2 production nor for IFNγ-production in either stimulation condition.

### Effect of protein expression on mixed chimerism

To investigate potential impact of protein expression on lymphocyte frequencies in patients with MC, correlation tests were undertaken between LCK/ZAP-70 expressions, percentages of cellular subsets and soluble characteristics. No significant correlations were found for ZAP-70 and tested parameters (i.e., cellular subsets, cytokine and immunoglobulin concentrations). Higher LCK expression was positively correlated with a shift towards a more differentiated T-cell phenotype for several T-cell subsets. The naïve memory T-cell frequency decreased and effector memory T-cell frequency increased with a higher LCK expression. This was significant in CD4+ T-cells (*P =* .001 and *P =* .011 respectively) and TCRαβ+ T-cells (*P =* .017 and *P =* .037 respectively). The other two differentiation subsets, central memory and terminally differentiated memory, were not correlated to LCK expression.

Finally, LCK expression was also correlated to an increased frequency of IL-2 and IFNγ-producing CD4+ T-cells (*P =* .026) and CD4+ T-cells producing only IFNγ (*P =* .006) after mitogenic stimulation. No correlation was observed for total T-cells or CD8+ T-cells.

### Chimerism status

**Fig 5:**
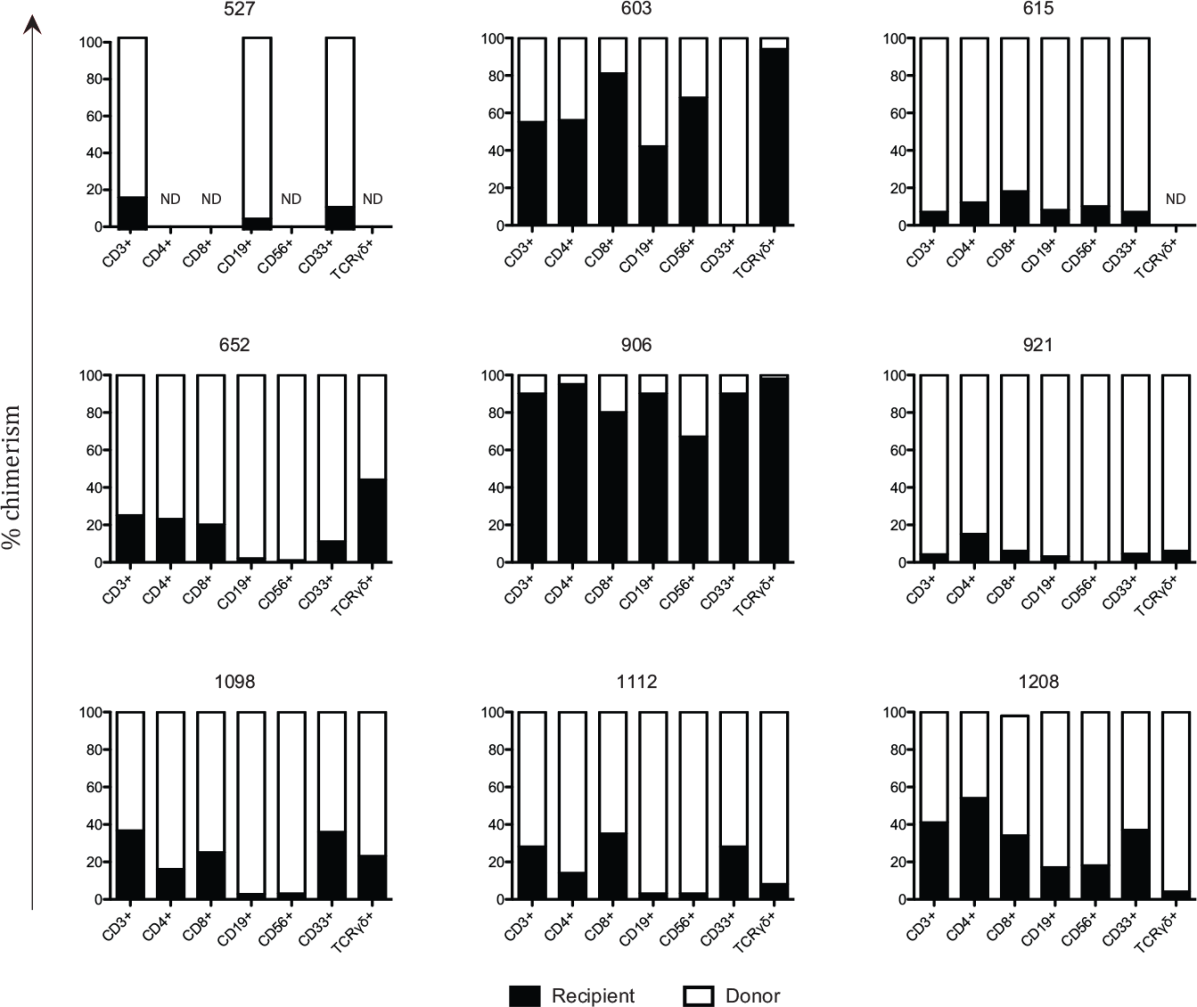
Recipient derived cells present in several cellular subsets in mixed chimerism patients. Each graph depicts percentages of chimerism for different cell subsets for each mixed chimerism (MC, n = 9) patient. Chimerism was analysed for T-cells (CD3+), CD4+ T-cells, CD8+ T-cells, B-cells (CD19+ CD3-), NK-cells (CD56+ CD3-), myeloid cells (CD33+) and TCRγδ+ T-cells. A black column represents the percentage of recipient derived cells, while the white column represents donor derived cells. ND depicts the subsets where chimerism analysis was unsuccessful due to insufficient DNA.

**Fig 6:**
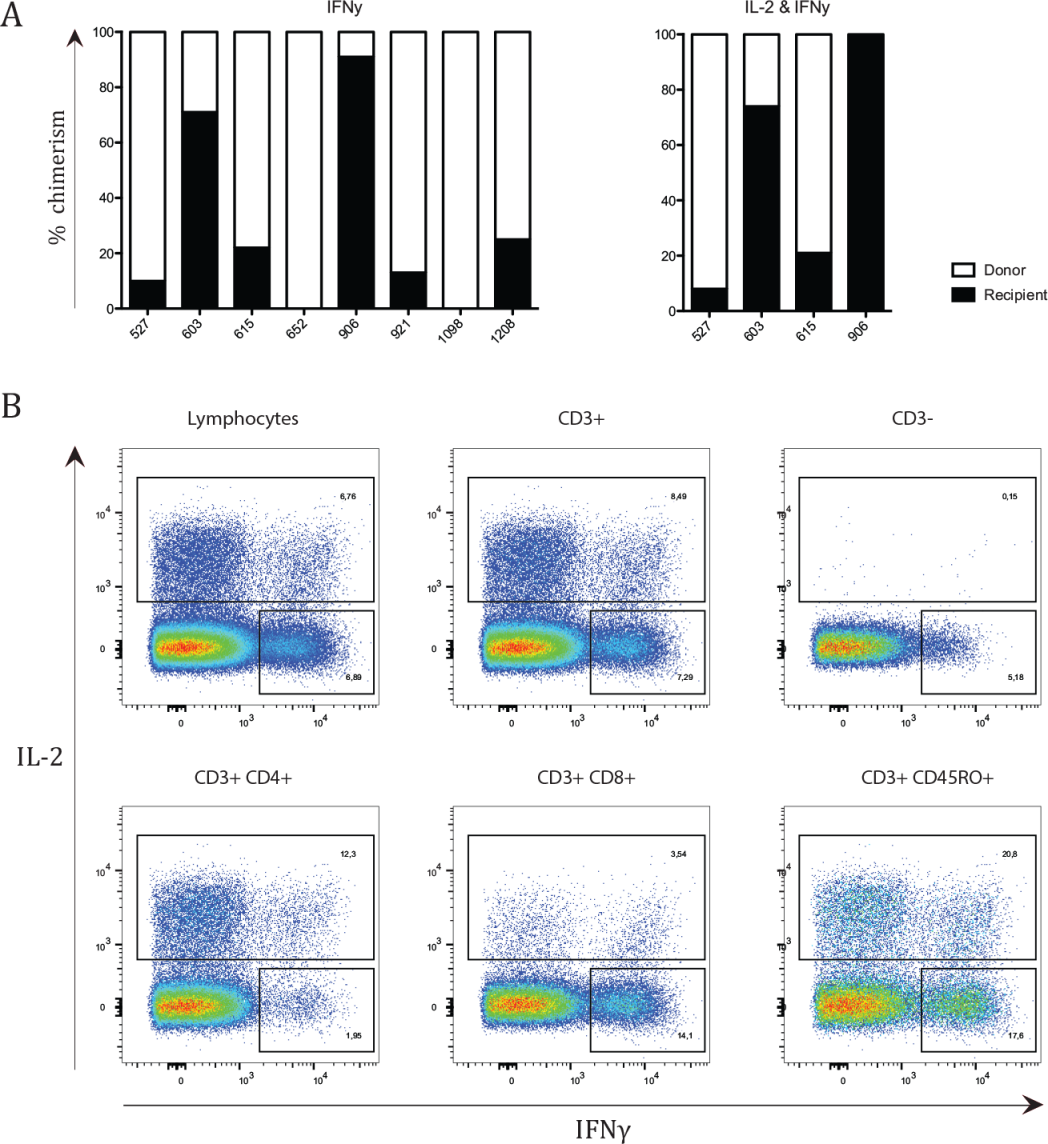
Recipient derived cells capable of cytokine production. (A) Each graph depicts percentages of chimerism for cytokine producing lymphocytes after a 4-hour incubation with PMA and Ionomycin. Chimerism was analysed for IFNγ-producing lymphocytes and IL-2 and IFNγ producing lymphocytes. A black column represents the percentage of recipient derived cells, while the white column represents donor-derived cells. ND depicts the subsets where chimerism analysis was unsuccessful due to insufficient DNA. (B) A representative plot from patient UPN 615 demonstrates the gating strategy used on varying cellular subsets. Sorting was done on total lymphocytes.

Due to few HLA mismatches between donor and recipient in patients with MC, it was not possible to separate donor and recipient system via flow cytometry. Instead cells were sorted by their phenotype and cytokine production and then separated for donor and recipient lineage via a chimerism analysis. Most patients with MC retained recipient-derived T-cells, B-cells, myeloid cells, NK-cells, CD4+ T-cells, CD8+ T-cells and TCRγδ+ T-cells (Fig 5). Additionally, recipient-derived cells responded to mitogenic stimuli by means of cytokine production (Fig 6). Supplementary S3 Fig displays a representative result of the chimerism analysis of patient UPN 906.

Despite the small sample size of the MC group, it is evident that the group seems to consist of two distinct groups; those with high percentage of recipient cells and those with low percentage of recipient cells (Figs 5 and 6). A division was made based on percentage of recipient derived T-cells. The group with low percentage of recipient T-cells consisted of patient 527, 615, 652, 921 and 1112, *e*.*g*. with a recipient:donor ratio of approximately 1:4. The group with high percentage of recipient T-cells consisted of patients 603, 906, 1098 and 1208, *e*.*g*. with a recipient:donor ratio of around 1:1. After analysing all phenotypic and functional markers, no differences were found between the patient groups except for levels of IgG3 and IL-12 (p40) at median 10 years post transplantation (*P =* .032 and *P =* .019 respectively). IgG3 levels were lower in patients with a high percentage of recipient T-cells (median of 0.76 μg/mL) than compared to patients with low percentage of recipient T-cells (median of 1.56 μg/mL). Patients with donor chimerism had a median IgG3 level of 0.8 μg/mL.

In contrast, IL-12 (p40) was higher in patients with a high percentage of recipient T-cells (median of 10.03 pg/mL) than compared to patients with low percentage of recipient T-cells (median of 6.29 pg/mL). Patients with donor chimerism had a median IL-12 (p40) level of 14 pg/mL.

## Discussion

Long-term stable MC is a rare and poorly understood phenomenon post-HSCT. We previously reported a potential mechanism driving MC after investigating chimerism patterns in a group of MC patients.[19] Here we focus on phenotypic and functional characterization of the immunologic features. Identification of putative differences compared to individuals with DC might help enlighten why some patients develop long-term stable MC post-HSCT. It might also shed light on the interplay between the two hematopoietic systems within MC patients. Is one system dominant over the other, or are both systems fully functional and active? Perhaps an even more important, clinical question; is long-term MC development disadvantageous for patients with non-malignant diseases? The answer could have an impact on future post-HSCT protocols as well as on patient’s future wellbeing.

In this study we investigated twelve MC and thirteen DC patients post-HSCT with non-malignant disease. The median follow-up was 10 years post-HSCT. This is a unique cohort, i.e., several patients with long-term stable MC treated at the same centre with a long follow-up time.

Even though blood stream infections were found to be more prevalent in patients with DC shortly post transplantation, as previously reported [19], no difference in infection incidence could be observed at median 10 years post transplantation. Additionally, there were no major differences regarding general wellbeing, cellular and soluble immune-phenotype between patients with MC and DC (Figs 1 and 2, S1 and S2 Figs and S3 Table), though some minor differences could be observed.

Patients with MC had higher IgG3 concentrations (Fig 1A). While high IgG3 concentrations have been correlated to ABO mismatching in solid organ transplantation [34], this was not the case for MC as all 5 MC patients with high IgG3 levels (above or at median) in this study were ABO-matched with their donor. Additionally, all had a high Karnofsky performance status (90 to 100) and none had clinically detectable autoimmune or allo-mediated diseases. Only two out of the five patients with high IgG3 had an unrelated and/or gender-mismatched donor, which would further speak against an obvious allo-mediated response between donor and recipient.[35, 36]

Plasma from patients with MC contained lower concentrations of IL-4, IL-12 (p40) and G-CSF (Fig 1B–1D). These findings could be due to a higher hematopoietic cell turnover with enhanced cytokine consumption in patients with MC.[37] While the higher turnover could be due to infections, the questionnaire demonstrated that patients with MC and DC had a similar infection burden, making this explanation unlikely. Therefore, it is more plausible that if patients with MC had a higher hematopoietic cell turnover it would stem from subclinical allo-reactive hematopoietic cells. However, none of the patients reported clinical allo-reactive like symptoms, which we could expect if self-antigen directed cells were active. Hence, it is unlikely that an increased hematopoietic cell turnover is the sole explanation of a lowered concentration of IL-4, IL-12 (p40) and G-CSF in MC patients.

An alternative explanation for the lowered concentration of IL-4, IL-12 (p40) and G-CSF is needed. One option is that patients with MC have a less inflammatory environment in the blood than patients with DC. This is indicated by the reduced concentration of pro-inflammatory cytokine IL-12 (p40) in patients with MC, which is important for NK-cell function and Th1 promotion.[38–40] G-CSF is known to stimulate granulopoiesis, but to suppress platelet production.[41, 42] Hence, lower G-CSF concentrations in patients with MC could reflect an increase in platelet counts, which was observed in these patients (Fig 2Aii). Finally, IL-4, known to promote the differentiation of Th2 cells via STAT6, was lower in patients with MC, indicating less differentiation towards Th2 cells.[43] A reduced Th2 phenotype opens up the possibility of a shift towards a Th1 phenotype. It has been shown that the Th1 cytokine IFNγ [44, 45] promotes the switch towards IgG3 [46], thus leading to higher concentrations of IgG3, as observed in the patients with MC (Fig 1A). However, a decrease in IL-12 (p40) would lead to less Th1 differentiation. Therefore, these two findings seemingly contradict each other, and more work is needed to resolve this.

To conclude, interpreting the results and the impact of the levels of IL-12 (p40), IL-4, G-CSF and IgG3 is difficult. Increasing the patient sample size in future studies will hopefully aid in elucidating the roles of these soluble markers.

Patients with MC and DC were similar in most hematopoietic cell subsets. One exception was the presence of a higher platelet count in the patients with MC (Fig 2Aii). However, due to the spread of the platelet count and the relatively small difference in median count (MC = 239 000 platelets/mL and DC = 198 000 platelets/mL) it is debatable whether this finding holds any clinical value. Interestingly, the three MC patients with the highest platelet count (UPN 527, 652 and 1112) were the same three with the highest IgG3 concentration in plasma. There was a positive correlation between IgG3 concentration and platelet count in the entire MC group (*P =* .05).

Another difference was that patients with MC had a higher frequency of potential NKT-cells (CD56+CD8+ and CD94+CD8+ T-cells) (Fig 2Diii and 2Dvi).[47] Unfortunately, no other NK-cell associated markers were included in the analysis at the same time, so it cannot be stated in definite terms that these subsets represent NKT-cells.[48, 49] It is also plausible that these cell subsets represent activated cytotoxic T-cells.[50] Data on the role of NKT-cells and inflammation is multi-faceted. The cells have both been shown to produce numerous pro-inflammatory cytokines upon activation, although other studies have shown that they can also shift to an anti-inflammatory response.[51]

In order to investigate the functionality of the lymphocytes, we measured protein expression of molecules important for the signalling cascade (Fig 3). One of the key proteins in the signalling cascade in T and NK-cells is the kinase ZAP-70.[52] Expression of ZAP-70 was lower in patients with MC (Fig 3B). In T-cells, the docking sites for ZAP-70 are facilitated by another kinase, LCK, which enables ZAP-70 to bind to CD3 ζ.[52] Expression of LCK and CD3 ζ (both predominantly expressed in T-cells) were similar in both patient groups. As only expression of ZAP-70 was lowered in patients with MC, it seems likely that ZAP-70 is a limiting factor in the signalling cascade for patients with MC. Therefore, it is possible that lymphocytes of these patients have a reduced potential for TCR signalling. A reduced level of proteins in the signalling cascade below the TCR has been described before and is associated with T-cell anergy.[53] This potential T-cell anergy would not be detectable by mitogenic stimulation (Fig 4A) as PMA/Ionomycin bypasses the TCR by directly activating Protein Kinase C.[54]

While LCK expression was similar for MC and DC patients; in MC patients, LCK expression was correlated to a shift towards a more differentiated phenotype. This was not observed in patients with DC. This could be partly due to the larger spread of LCK expression in MC patients (Fig 3B).

Additionally, LCK expression was correlated to an increased frequency of IL-2 producing CD4+ T-cells after stimulation. While inconclusive, it is striking that MC patients with a high LCK expression also have a high frequency of IL-2 producing cells in multiple cell subsets; implying an overall link between LCK expression and IL-2 production.

Both patients with MC and DC had similar frequencies of IL-2 and IFNγ producing cells in several T-cell subsets after mitogenic stimulation (Fig 4A). Despite this, lymphocytes derived from patients with MC had higher frequencies of IL-2 producing T-cells in the non-stimulated control samples (Fig 4B and 4C). As the non-stimulated condition could be considered an indicator of steady state cytokine production, it is possible that patients with MC have a higher frequency of steady state IL-2 producing lymphocytes.

IL-2 is known to promote differentiation of T-cells into regulatory T-cells and/or promote T-cells towards an effector memory phenotype.[55] In line with this, as described above, we identified a positive correlation between LCK expression and a shift towards a more differentiated effector memory phenotype as well as a correlation between LCK expression and IL-2 producing CD4+T-cells in patients with MC, but not DC. No difference was found between the patients regarding the regulatory and effector memory T-cell subsets frequencies.

We previously reported that chimerism patterns for patients with MC fluctuated primarily during the first couple of years post-HSCT, stabilizing after several years. Additionally, it seemed that recipient chimerism of the T, B and myeloid cell-lineages tended to follow each other.[19] Here, we further assessed chimerism status for additional cell subsets; NK-cells, CD4+ T-cells, CD8+ T-cell, TCRγδ+ T-cells and cytokine-producing lymphocytes. Almost all patients with MC retained recipient cells in these subsets (Figs 5 and 6A). The discovery of recipient-derived cells among cytokine-producing lymphocytes shows that recipient cells are still able to respond to mitogenic stimuli. This is consistent with the fact that there was no difference in antibody concentrations against antigens from the patient’s re-immunizations post-HSCT (S1E–S1H Fig), even in patients with high recipient chimerism. It is unclear whether the recipient and donor system respond to the same *in vivo* stimuli or whether the recipient and donor system found niches within the immune system. If the latter is true, the separate niches must consist of small subsets as all of the major subsets (*e*.*g*., B-cells, NK-cells, CD4+ T-cells, CD8+ T-cell, TCRγδ+ T and myeloid cells) consist of both recipient- and donor-derived hematopoietic cells. Stimulating lymphocytes from patients with MC with antigen-specific stimuli *in vitro* could potentially elucidate this. Unfortunately, due to the limited amount of samples, it was not possible to do such an experiment within the scope of this study.

Lastly, we attempted to elucidate whether the heterogeneity of percentage of recipient T-cells in patients with MC had an effect on immune-phenotype. The MC group was split into 2 groups, one group of 4 patients who were high in percentage of recipient T-cells (1:1 recipient:donor ratio) and one group of 5 patients who were low in percentage of recipient T-cells (1:4 recipient:donor ratio). IgG3 and IL-12 (p40) levels were found to differ between these two MC groups. Interestingly, the MC patients with a 1:1 recipient:donor ratio had IgG3 and IL-12 (p40) levels more similar to the donor chimerism setting than the MC patients with a 1:4 recipient:donor ratio did. However, as these two subgroups were extremely small (4 against 5) we should be cautious before we ascribe conclusions to these results. A larger group size could better ascertain whether these differences between the 2 MC groups are true differences or a matter of statistical spread.

In conclusion; here we show that patients with long-term stable MC are in good health and appear to be immunologically similar to patients with DC. Patients with MC may actually have a less inflammatory environment than DC patients, suggesting development of tolerance between the donor- and recipient-derived hematopoietic systems. Interestingly, and opposed to a similar study on donor-donor chimerism after DCBT [18], it appears that both donor and recipient hematopoietic systems are active and functional within the patients with MC. These results raise the question of whether it is necessary to use DLIs to combat emerging MC in individuals with non-malignant disorders. If the MC remains stable and does not progress, DLI treatment is perhaps not worth the risk of GvHD for both the patient and the clinic.

## Supporting Information

S1 Fig Comparison of IgG concentration and vaccination titres against common bacteria in plasma between patients with mixed and donor chimerism. (A-D) Concentrations of IgG and IgG subclasses were determined in plasma of 9 mixed chimerism (MC) and 10 donor chimerism (DC) patients. (A) Total IgG, (B) IgG1, (C) IgG2 and (D) IgG4 concentrations in plasma for DC and MC patients. No difference was observed. (E-H) Vaccination antibody titres for *C*. *Diptheriae* (E), *C*. *Tetani* (F), *S*. *Pneunomiae* (G) and H. Influenzae (H) are shown for DC and MC patients. No difference was observed between the MC and DC patient groups. IE = International Unit. Symbols indicate individual patient levels and horizontal bars in scatter graphs indicate median values of the patient group.
(PDF)

S2 Fig Phenotypic comparison of NK, B, CD4 and CD8 T cell subsets between patients with mixed and donor chimerism. For most cellular subsets no significant differences were observed between 9 mixed chimerism (MC) and 10 donor chimerism (DC) patients. (A) Representative NK-cell (CD56+CD3-; i-ii) and B-cell (CD19+CD3-; iv-v) FACS plots from both patient groups. The corresponding graph shows the individual percentages of NK (iii) and B-cells (vi) in the patient groups. (B) Representative FACS plots of CD4+ and CD8+ cells gated on CD3+ lymphocytes (i-ii). The accompanying graph depicts no difference in individual percentages of the CD4/CD8 ratio between the groups (iii).
(PDF)

S3 Fig Representative chimerism analysis of MC patient. Chimerism analysis of patient UPN 906. The first two panels (i-ii) show the distinctive peaks for the patient’s and donor’s DNA. Subsequently, the next 9 graphs (iii-xi) demonstrate the peaks for each cell subset.
(PDF)

S1 Table Methods. MC = Mixed Chimerism; DC = Donor Chimerism; UPN = Unique Patient Number; ELISA = Enzyme Linked Immuno Sorbent Assay; FACS = Fluorescence Activated Cell Sorting; WB = Western Blot; * = chimerism was only assessed for CD3, CD19 and CD33 cell lineages
(DOCX)

S2 Table Questionnaire results. n = Number of patients
(DOCX)

S3 Table Soluble biomarkers. HSCT = Hematopoietic Stem Cell Transplantation; MC = Mixed Chimerism; DC = Donor Chimerism; G-CSF = Granulocyte Colony-Stimulating Factor; IFN = Interferon; IL = Interleukin; Ig = Immunoglobulin
(DOCX)
