# Supplementary figures and images for: Long-Term Stable Mixed Chimerism after Hematopoietic Stem Cell Transplantation in Patients with Non-Malignant Disease, Shall We Be Tolerant?
Source: PLoS One. 2016 May 6;11(5):e0154737. doi: 10.1371/journal.pone.0154737 (PMC4859543; doi:10.1371/journal.pone.0154737)

# S1 Figure

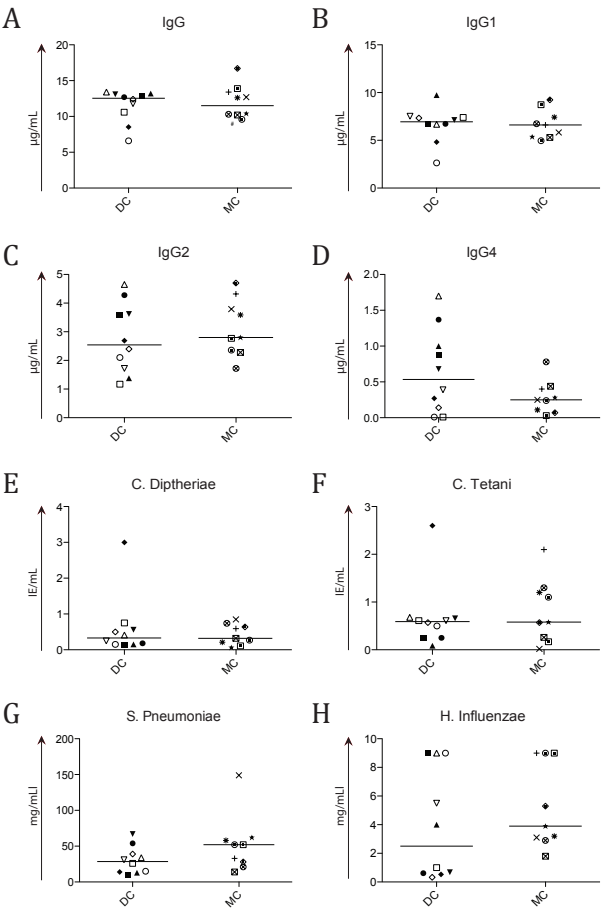

Supplement: S1 Fig — (A-D) Concentrations of IgG and IgG subclasses were determined in plasma of 9 mixed chimerism (MC) and 10 donor chimerism (DC) patients. (A) Total IgG, (B) IgG1, (C) IgG2 and (D) IgG4 concentrations in plasma for DC and MC patients. No difference was observed. (E-H) Vaccination antibody titres for C. Diptheriae (E), C. Tetani (F), S. Pneunomiae (G) and H. Influenzae (H) are shown for DC and MC patients. No difference was observed between the MC and DC patient groups. IE = International Unit. Symbols indicate individual patient levels and horizontal bars in scatter graphs indicate median values of the patient group. (PDF) [file pone.0154737.s001.pdf]

S2 Figure

A

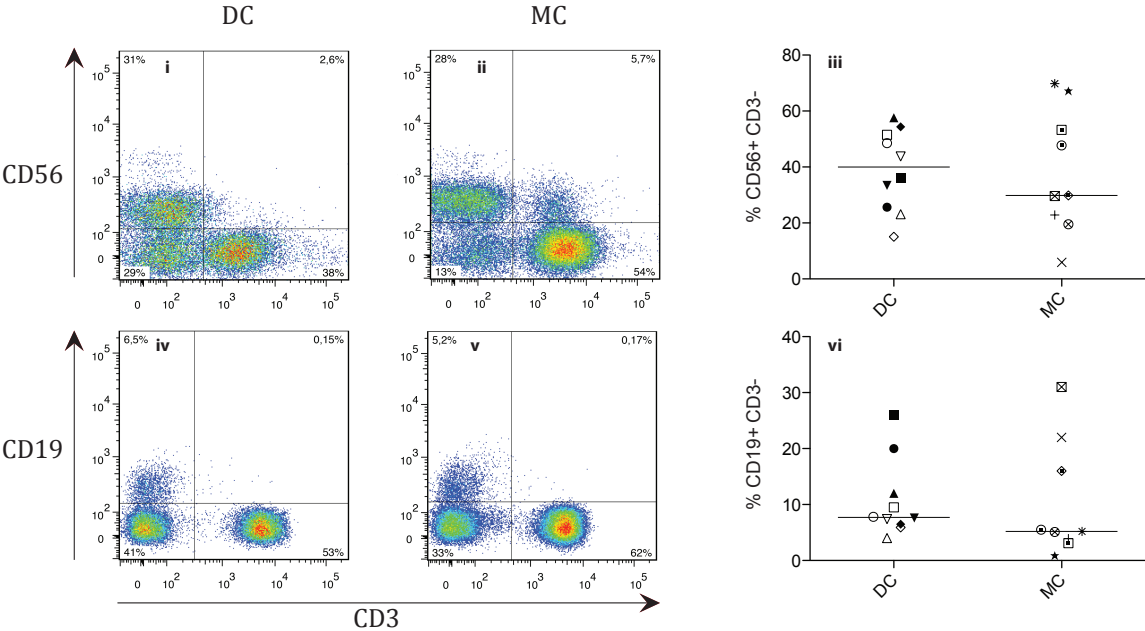

B

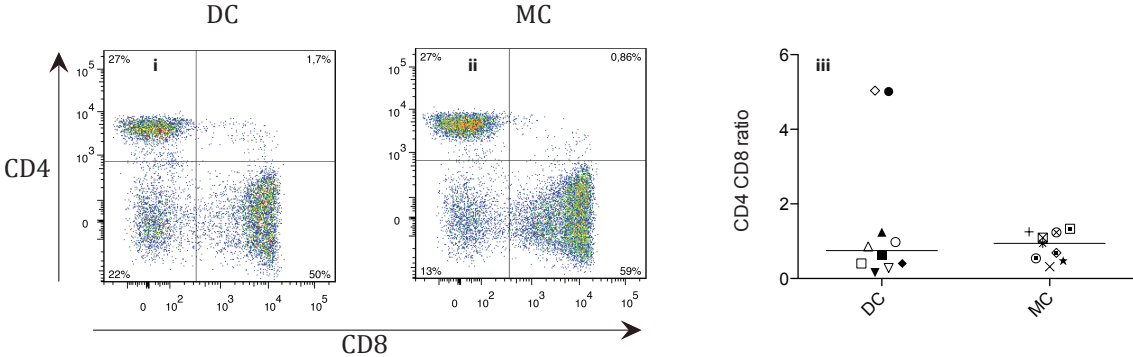

Supplement: S2 Fig — For most cellular subsets no significant differences were observed between 9 mixed chimerism (MC) and 10 donor chimerism (DC) patients. (A) Representative NK-cell (CD56+CD3-; i-ii) and B-cell (CD19+CD3-; iv-v) FACS plots from both patient groups. The corresponding graph shows the individual percentages of NK (iii) and B-cells (vi) in the patient groups. (B) Representative FACS plots of CD4+ and CD8+ cells gated on CD3+ lymphocytes (i-ii). The accompanying graph depicts no difference in individual percentages of the CD4/CD8 ratio between the groups (iii). (PDF) [file pone.0154737.s002.pdf]

# S3 Figure

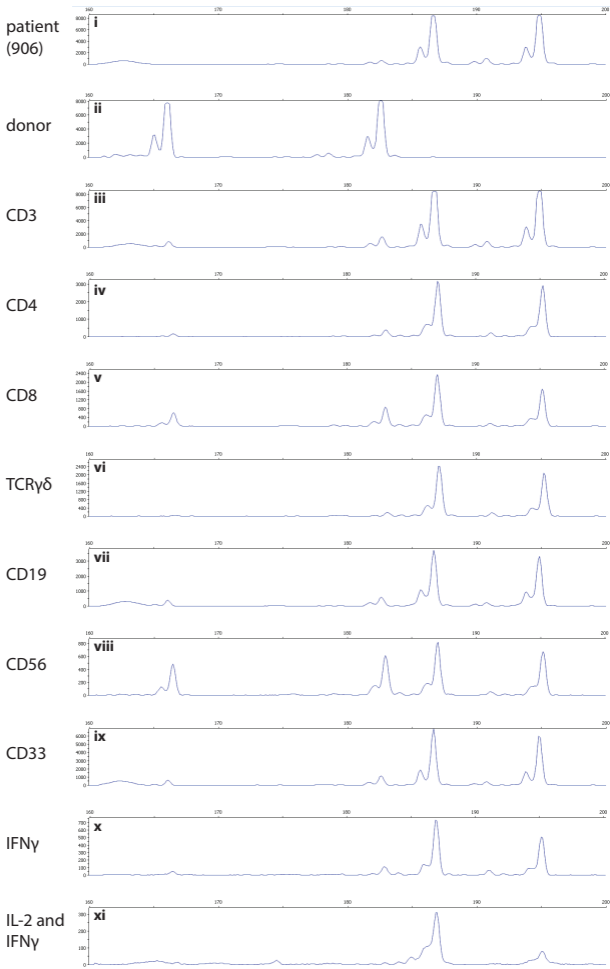

Supplement: S3 Fig — Chimerism analysis of patient UPN 906. The first two panels (i-ii) show the distinctive peaks for the patient’s and donor’s DNA. Subsequently, the next 9 graphs (iii-xi) demonstrate the peaks for each cell subset. (PDF) [file pone.0154737.s003.pdf]
