# Supplementary material for: Long-Term Stable Mixed Chimerism after Hematopoietic Stem Cell Transplantation in Patients with Non-Malignant Disease, Shall We Be Tolerant?
Source: PLoS One. 2016 May 6;11(5):e0154737. doi: 10.1371/journal.pone.0154737 (PMC4859543; doi:10.1371/journal.pone.0154737)
Supplement: S1 Table — MC = Mixed Chimerism; DC = Donor Chimerism; UPN = Unique Patient Number; ELISA = Enzyme Linked Immuno Sorbent Assay; FACS = Fluorescence Activated Cell Sorting; WB = Western Blot; * = chimerism was only assessed for CD3, CD19 and CD33 cell lineages (DOCX) [file pone.0154737.s004.docx]

**S1 Table.**

| **Chimerism status** | **UPN** | **Questionnaires** | **Chimerism** | **HLA typing** | **ELISA** | **Immuno-nephelometry** | **FACS** | **Multiplex day 14** | **Multiplex**  **>5years** | **Western Blot** | **Mitogenic Stimulation** |
| --- | --- | --- | --- | --- | --- | --- | --- | --- | --- | --- | --- |
| **MC** | 527 | x | x | x | x | x | x |  | x | x | x |
|  | 539 | x | x* | x |  |  |  |  |  |  |  |
|  | 603 | x | x | x | x | x | x |  | x | x | x |
|  | 615 | x | x | x | x | x | x |  | x | x | x |
|  | 652 | x | x | x | x | x | x |  | x | x | x |
|  | 906 | x | x | x | x | x | x | x | x | x | x |
|  | 921 | x | x | x | x | x | x |  | x | x | x |
|  | 1012 | x | x* | x |  |  |  |  |  |  |  |
|  | 1098 | x | x | x | x | x | x |  | x | x | x |
|  | 1112 | x | x | x | x | x | x | x | x | x | x |
|  | 1208 | x | x | x | x | x | x | x | x | x | x |
|  | 1240 | x | x* | x |  | x |  | x |  |  |  |
| **DC** | 628 | x | x* | x | x | x | x |  | x | x | x |
|  | 707b | x | x* | x | x | x | x |  | x | x | x |
|  | 731 | x | x* | x | x | x | x |  | x | x | x |
|  | 822 | x | x* | x |  |  |  | x |  |  |  |
|  | 887 | x | x* | x | x | x | x | x | x | x | x |
|  | 909 | x | x* | x | x | x | x | x | x | x | x |
|  | 954 |  | x* | x |  |  |  | x |  |  |  |
|  | 955 | x | x* | x | x | x | x | x | x | x | x |
|  | 1065 | x | x* | x | x | x | x | x | x | x | x |
|  | 1111 |  | x* | x |  |  |  | x |  |  |  |
|  | 1166 | x | x* | x | x | x | x | x | x | x | x |
|  | 1167 | x | x* | x | x | x | x |  | x | x | x |
|  | 1229 | x | x* | x | x | x | x |  | x | x | x |
