# Supplementary material for: Long-Term Stable Mixed Chimerism after Hematopoietic Stem Cell Transplantation in Patients with Non-Malignant Disease, Shall We Be Tolerant?
Source: PLoS One. 2016 May 6;11(5):e0154737. doi: 10.1371/journal.pone.0154737 (PMC4859543; doi:10.1371/journal.pone.0154737)
Supplement: S2 Table — n = Number of patients (DOCX) [file pone.0154737.s005.docx]

**S2 Table.**

|  | **Donor chimerism**  **(n=11)** | **Mixed chimerism**  **(n=12)** |
| --- | --- | --- |
| **Diarrhea or loose stool (no / yes / unknown)** | 8 / 2 / 1 | 10 / 2 / 0 |
| **Fever without reasonable explanation (no / yes)** | 9 / 2 | 10 / 2 |
| **Infections (no / yes / unknown)**  **Ear infection**  **Sinus infection**  **Bronchitis**  **Pneumonia** | 6 / 4 / 1  8 / 3 / 0  8 / 2 / 1  7 / 4 / 0 | 9 / 3 / 0  11 / 1 / 0  10 / 2 / 0  9 / 3 / 0 |
| **Skin problems (no / yes)** | 4 / 7 | 7 / 5 |
| **Fulltime job or study (no / yes)** | 4 / 7 | 4 / 8 |
| **Medication usage (no / yes)**  **Immunosuppressive**  **Calcium and osteoporosis**  **Heart pressure**  **Mental (depression, schizophrenia, anxiety)**  **Hormonal (thyroid, diabetes)** | 10 / 1  10 / 1  10 / 1  10 / 1  8 / 3 | 10 / 2  11 / 1  11 / 1  10 / 2  9 / 3 |
| **Sick leave days per year (0 / 1-8 / 8-15 / >15 / unknown)** | 6 / 3 / 1 / 0 / 1 | 3 / 5 / 0 / 3 / 1 |
| **Antibiotic regimens during last 5 years (0 / 2-4 / >5)** | 2 / 7 / 2 | 6 / 4 / 2 |
