# Supplementary material for: Long-Term Stable Mixed Chimerism after Hematopoietic Stem Cell Transplantation in Patients with Non-Malignant Disease, Shall We Be Tolerant?
Source: PLoS One. 2016 May 6;11(5):e0154737. doi: 10.1371/journal.pone.0154737 (PMC4859543; doi:10.1371/journal.pone.0154737)
Supplement: S3 Table — HSCT = Hematopoietic Stem Cell Transplantation; MC = Mixed Chimerism; DC = Donor Chimerism; G-CSF = Granulocyte Colony-Stimulating Factor; IFN = Interferon; IL = Interleukin; Ig = Immunoglobulin (DOCX) [file pone.0154737.s006.docx]

**S3 Table.**

| **Sample taken** | **Marker** | **Median concentration DC patients (range)** | | **Median concentration MC patients (range)** | | ***P*** |
| --- | --- | --- | --- | --- | --- | --- |
| **Day 14** | G-CSF | 203.96 | (20.85 - 3362) | 383.77 | (17.18 - 893.81) | *.808* |
|  | IFNγ | 7.1 | (2.12 - 17.24) | 10.24 | (2.12 - 44.68) | *.610* |
|  | IL-1a | 2.26 | (2.26 - 12.3) | 4.69 | (2.26 - 10.08) | *.613* |
|  | IL-4 | 2.31 | (2.31 - 3.47) | 3.88 | (2.31 - 16.14) | *.088* |
|  | IL-10 | 6.17 | (2.63 - 44.25) | 2.63 | (2.63 - 4.83) | *.064* |
|  | IL-12 (p40) | 2.47 | (2.47 - 16.03) | 2.47 | (2.47 - 2.98) | *.544* |
|  | IL-17 | 2.95 | (2.95 - 2.95) | 2.95 | (2.95 - 5.42) | *.216* |
| **>5 years** | G-CSF | 13.3 | (2.98 - 30) | 2.98 | (0.3 - 34.89) | ***.022*** |
|  | IFNγ | 3.12 | (0.48 - 19.57) | 1.29 | (0.35 - 5.2) | *.094* |
|  | IL-1a | 28.91 | (7.69 - 139) | 35.78 | (20.06 - 53.5) | *1.000* |
|  | IL-4 | 0.16 | (0.02 - 9.17) | 0.01 | (0 - 0.22) | ***.016*** |
|  | IL-10 | 6.12 | (0.71 - 14.05) | 7.26 | (2.99 - 11.9) | *.347* |
|  | IL-12 (p40) | 13.69 | (8.15 - 52.17) | 7.26 | (1.94 - 11.9) | ***.003*** |
|  | IL-17 | 2.17 | (0.51 - 11.9) | 1.68 | (0.62 - 3.05) | *.307* |
|  | IgG | 12.55 | (6.59 - 13.4) | 11.5 | (9.59 - 16.7) | *.970* |
|  | IgG1 | 6.95 | (2.64 - 9.74) | 6.61 | (4.96 - 9.24) | *.720* |
|  | IgG2 | 2.55 | (1.17 - 4.65) | 2.8 | (1.72 - 4.7) | *.390* |
|  | IgG3 | 0.80 | (0.27 - 0.92) | 1.25 | (0.45 - 1.8) | ***.027*** |
|  | IgG4 | 0.39 | (0.01 - 1.7) | 0.25 | (0.03 - 0.78) | *.307* |
|  | *C. Diptheriae* (IgG) | 0.33 | (0.13 - 3) | 0.32 | (0.06 - 0.85) | *.903* |
|  | *C. Tetani* (IgG) | 0.59 | (0.08 - 2.6) | 0.58 | (0.02 - 2.1) | *.744* |
|  | *S. Pneumoniae* (IgG) | 28.5 | (9.3 - 67) | 52 | (14 - 149) | *.362* |
|  | *H. Influenzae* (IgG) | 2.5 | (0.33 - 9) | 3.9 | (1.8 - 9) | *.191* |
